# Sublethal doses of imidacloprid disrupt sexual communication and host finding in a parasitoid wasp

**DOI:** 10.1038/srep42756

**Published:** 2017-02-15

**Authors:** Lars Tappert, Tamara Pokorny, John Hofferberth, Joachim Ruther

**Affiliations:** 1 Institute of Zoology, University of Regensburg, 93053 Regensburg, Germany; 2 Department of Chemistry, Kenyon College, Gambier, OH 43022, USA

## Abstract

Neonicotinoids are widely used insecticides, but their use is subject of debate because of their detrimental effects on pollinators. Little is known about the effect of neonicotinoids on other beneficial insects such as parasitoid wasps, which serve as natural enemies and are crucial for ecosystem functioning. Here we show that sublethal doses of the neonicotinoid imidacloprid impair sexual communication and host finding in the parasitoid wasp *Nasonia vitripennis.* Depending on the dose, treated females were less responsive to the male sex pheromone or unable to use it as a cue at all. Courtship behaviour of treated couples was also impeded resulting in a reduction of mating rates by up to 80%. Moreover, treated females were no longer able to locate hosts by using olfactory cues. Olfaction is crucial for the reproductive success of parasitoid wasps. Hence, sublethal doses of neonicotinoids might compromise the function of parasitoid wasps as natural enemies with potentially dire consequences for ecosystem services.

The use of insecticides is a relatively cheap and effective way to control insect pests, but it poses ecological problems by also affecting non-target organisms^1^. This is particularly true for chemically stable broad-spectrum insecticides that might persist in the environment and accumulate in food chains. Apart from directly causing mortality or malformation, insecticides may also harm insects at sublethal doses, for instance after the consumption of contaminated pollen and nectar^2^. Since many classes of insecticides are neurotoxins, they can have serious effects on the insects’ sensory system and thus impede their ability to acquire information from their environment^3^,^4^. Olfaction is of particular importance for reproduction and survival of most insect species^5^. Hence, the disruption of olfaction by sublethal doses of insecticides might have severe consequences for the fitness of insects and severely compromise their contribution to ecosystem services^3^.

Worldwide, the most applied insecticides are the neonicotinoids^6^,^7^,^8^. They act selectively on the insect central nervous system by binding competitively to nicotinergic acetylcholine receptors^9^. This causes a depolarization block of neuronal firing and inhibits nicotinergic responses even at sublethal doses, which results in an uncontrolled continuous stimulation of the respective neurons and finally the death of the animal. The economic success of neonicotinoids is due to their high effectivity and relatively low toxicity for mammals^9^. The development of resistances by many insect pests to other classes of insecticides additionally fostered the success of neonictinoids and resulted in a global market share of 28.5%^7^. Neonicotinoids are typically used as seed coatings^10^,^11^ and act systemically, i.e., after germination, the pesticides are absorbed by the roots and transported via the xylem to the aboveground parts of the plant. They can be detected not only in the plant tissue but also in guttation drops^12^, nectar and pollen^10^,^13^ and are thus, accessible to non-target insects^14^. Because of their massive use in agriculture and a relatively high stability, neonicotinoids are persistent in the environment and occur even in ground water^14^.

There is ample literature indicating detrimental effects of neonicotinoids on pollinators^1^,^15^. In honeybees, sublethal doses of neonicotinoids impair orientation behaviour and foraging efficiency and cause increased mortality due to homing failure^16^,^17^,^18^,^19^. Neonicotinoid treated honeybee workers show decreased sucrose responses (proboscis extension reflex) and are less active in performing “waggle dance”, an important component of forager recruitment^20^. Furthermore, neonicotinoids have negative effects on the immune system of honeybees^21^. In bumblebees, exposure to sublethal doses of neonicotinoids decrease foraging efficiency, fecundity and colony performance^22^,^23^,^24^. A recent field study has revealed that coating of oilseed rape seeds with a combination of neonicotinoids and pyrethroids reduces wild bee abundance, solitary bee nesting, as well as bumblebee colony growth and reproduction^10^. Therefore, the extensive use of neonicotinoids is discussed as an important factor contributing to the currently observed decline of wild bee diversity and abundance^21^,^23^. As a consequence, the European Union has decreed in 2013 a moratorium on the three most purchased neonicotinoids imidacloprid, chlothianidin and thiamethoxam as seed coatings for crops that attract bees^25^.

Besides pollinators, natural enemies of insect pests such as parasitoid wasps also perform key ecosystem services^26^,^27^. Larvae of parasitoid wasps develop in or on the body of other arthropods and kill their host at the end of development. Hence, parasitoid wasps are indispensable as natural enemies and are used for biological control of insect pests^27^,^28^,^29^. Many species of parasitoid wasps visit flowers and feed on nectar and pollen^30^. Thus parasitoid wasps are, like bees, at risk of ingesting neonicotinoids and other systemic pesticides. It has been shown that the uptake of nectar containing neonicotinoids may impair the host finding ability of females^31^, but little is known about the sublethal effects of neonicotinoids on the sexual communication of parasitoid wasps.

In the present study, we investigate the effects of sublethal doses of imidacloprid on sexual communication and olfactory host finding in the jewel wasp *Nasonia vitripennis,* which parasitizes the pupae of several fly species. Pheromones are involved at different levels of sexual communication in *N. vitripennis*. Males produce in their rectum a mixture of (4 *R*,5 *R*)- and (4 *R*,5 *S*)-5-hydroxy-4-decanolides (HDL) and 4-methylquinazoline that is highly attractive to virgin females^32^,^33^. Males recognise potential mates by female-derived cuticular hydrocarbons that elicit stereotypic courtship behaviour^34^,^35^,^36^. After recognition of a female, the male mounts the female and performs precopulatory courtship involving a characteristic “head nodding” behaviour that serves the release of a yet unidentified aphrodisiac pheromone, which is essential for the female to signal receptivity^34^,^37^. The female signals receptivity by lowering her head and antennae and simultaneously opening her genital orifice. Subsequently, the male establishes genital contact and copulates with the female. After copulation, the male remounts the female and performs postcopulatory courtship that consists of the same elements as the precopulatory courtship^34^,^38^,^39^. After mating, the female is no longer attracted to the male pheromone and begins to search for a suitable host patch for oviposition^32^,^37^,^40^. Olfactory stimuli are also of crucial importance for host selection^41^,^42^.

In the present study, we investigate the toxic effects of externally applied imidacloprid for *N. vitripennis* wasps and, using standardized olfactometer bioassays, demonstrate that sublethal imidacloprid doses impede the ability of *N. vitripennis* females to orient toward both the male sex pheromone and host odour. Furthermore, we show that the courtship behaviour of *N. vitripennis* is impaired and mating rates are reduced if one or both partners are treated with sublethal doses of imidacloprid.

## Results

### Determination of LD_50_

**Figure 1:**
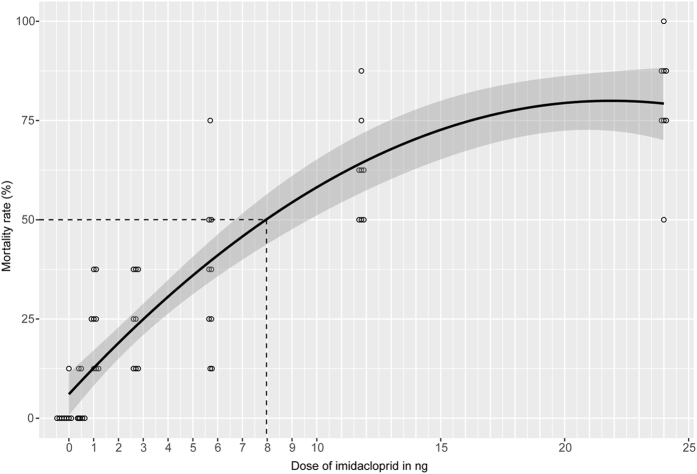
Toxicity of externally applied imidacloprid for *N. vitripennis*. Mortality rates after 72 h of *N. vitripennis* treated with different doses of the neonicotinoid imidacloprid dissolved in acetone (n = 8 replicates with 8 wasps each; overlaying data points for each dose were manually shifted horizontally for clarity). The 95% confidence interval is indicated in darker grey. The dashed line indicates the LD_50_.

Most previous studies on the effects of neonicotinoids on non-target insects offered the active substance with food^17^,^18^,^21^,^22^,^23^,^24^,^43^,^44^,^45^,^46^,^47^,^48^. This approach, however, does not allow for the accurate quantification of the incorporated dose, because the amount of food consumed is difficult to determine. Therefore, we applied imidacloprid as acetone solutions to the abdominal tip of cold-sedated *N. vitripennis.* With this technique, the dose causing 50% mortality (LD_50_) within a 72 h observation period was 8 ng per wasp (Fig. 1). In the bioassays, we used doses between 0.1 and 1.1 ng per wasp (1.25–13.75% of the LD_50_) to study the effects of sublethal imidacloprid doses on the sexual communication and host finding in *N. vitripennis*.

### Effect of sublethal imidacloprid doses on female response to the male sex pheromone

**Figure 2:**
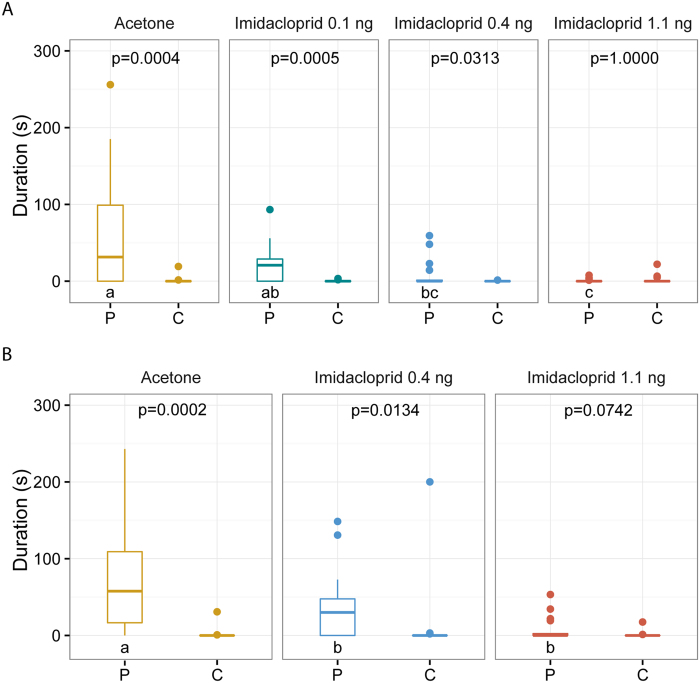
Effect of sublethal doses of imidacloprid on the response of virgin *N. vitripennis* females to the male sex pheromone. Females were treated with solutions of imidacloprid in acetone or pure solvent (control) and tested (**A**) one or (**B**) three days after the treatment in a two-choice olfactometer. Given here are the durations females stayed in odour fields treated with the pheromone (P) or the pure solvent (C, control) during an observation time of five minutes. Box-and-whisker plots show median (horizontal line), 25–75% quartiles (box), maximum/minimum range (whiskers) and outliers (>1.5 × above box height). Statistical analysis for each treatment by Wilcoxon matched pairs test (n = 20). Comparisons between treatments by Kruskal-Wallis *H-*test (data given in the results) followed by multiple pairwise Mann-Whitney *U*-tests with sequential Bonferroni correction. Different lowercase letters within each panel indicate significant differences at p < 0.05.

We treated virgin females with acetone (control), 0.1, 0.4 or 1.1 ng of imidacloprid and tested them 1 d after the treatment in a well-established two-choice olfactometer test^49^. Treated females were given the choice between synthetic male sex pheromone and pure solvent. The attraction of virgin females to the male sex pheromone decreased with increasing imidacloprid dose (Kruskal-Wallis test: H = 15.18; p < 0.001, Fig. 2A) and females treated with 1.1 ng no longer discriminated between the male pheromone and the solvent control. This effect persisted 3 d after the treatment (Kruskal-Wallis test: H = 14.71; p < 0.001, Fig. 2B).

### Effect of sublethal imidacloprid doses on female response to host odour

**Figure 3:**
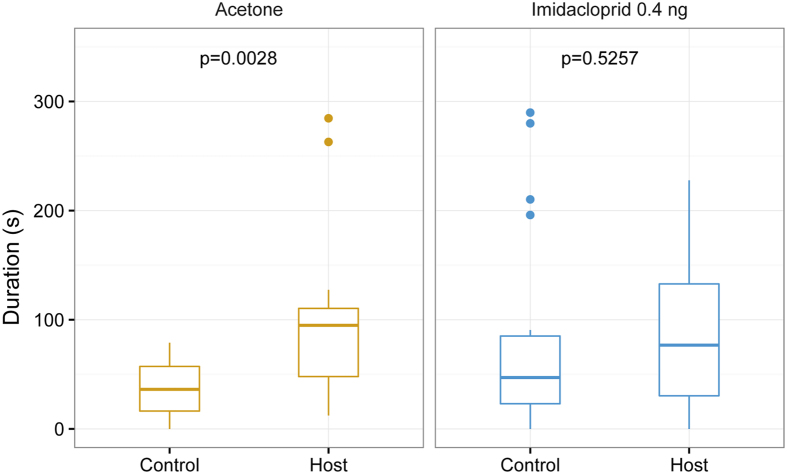
Effect of a sublethal dose of imidacloprid on the response of *N. vitripennis* females to host odour. Females were treated with a solution of imidacloprid in acetone or the pure solvent (control) and tested in a two-choice-olfactometer. Given here are the durations females stayed in one of two odour fields scented with either five pupae of the green bottle fly, *Lucilia caesar*, or left unscented (control) during an observation time of five minutes. Box-and-whisker plots show median (horizontal line), 25–75% quartiles (box), maximum/minimum range (whiskers) and outliers (>1.5 × above box height). Statistical analysis by Wilcoxon matched pairs test (n = 20).

In a bioassay using another type of still-air olfactometer^40^ mated females were given the choice between the odour of five host puparia and an unscented control. While acetone-treated control females were attracted to the host odour, females treated with 0.4 ng imidacloprid no longer preferred the volatile host cues over the unscented control (Fig. 3).

### Effect of sublethal imidacloprid doses on courtship behaviour

**Figure 4:**
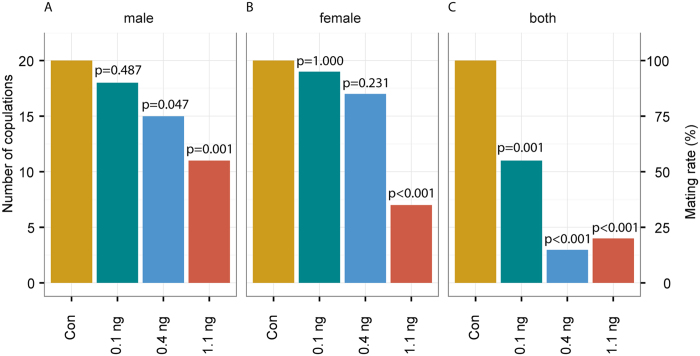
Effect of sublethal doses of imidacloprid on the mating rate of *N. vitripennis*. Mating rates of *N. vitripennis* couples with (**A**) the male, (**B**) the female or (**C**) both partners treated with a solution of imidacloprid in acetone or pure solvent (control). P-values refer to comparisons between different imidacloprid doses and the control mating rate (=100%; statistical analysis by Fisher’s exact test, n = 20 for each experiment; Freeman-Halton test across treatments significant for all panels at p < 0.001).

**Figure 5:**
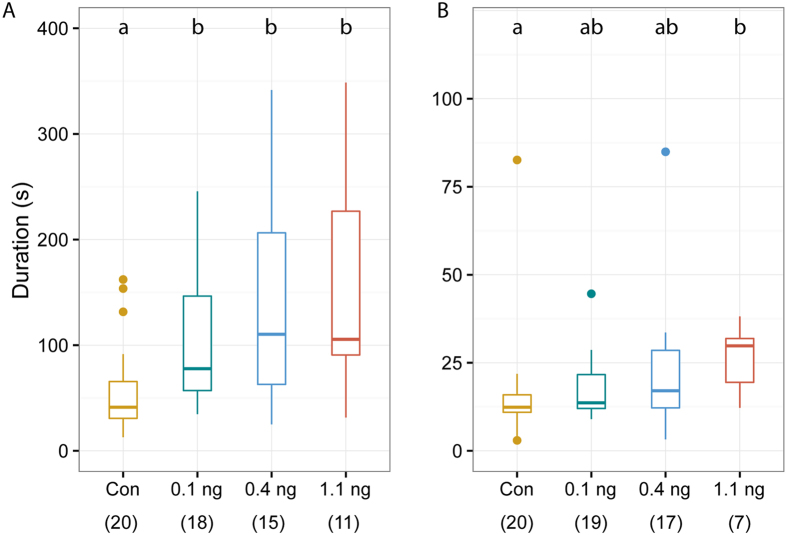
Effect of sublethal doses of imidacloprid on the courtship behaviour of *N. vitripennis*. (**A**) Time between start of the observation and copulation in couples with treated males. (**B**) Time between ‘male mounts the female’ and ‘female shows receptivity signal’ in couples with treated females. Wasps were treated with a solution of imidacloprid in acetone or the pure solvent (Con). Numbers of replicates (given in parentheses) differ because only those couples that started courtship within 5 min were included. Box-and-whisker plots show median (horizontal line), 25–75% quartiles (box), maximum/minimum range (whiskers) and outliers (>1.5 × above box height). Different lowercase letters indicate significant differences at p < 0.05; statistical analysis by Kruskal-Wallis test (data given in the results) followed by multiple pairwise Mann-Whitney *U*-tests with sequential Bonferroni correction.

To study the sublethal effects of imidacloprid on the courtship behaviour of *N. vitripennis*, we observed couples in which the male, the female or both partners had been treated with acetone (control), 0.1, 0.4 or 1.1 ng imidacloprid. If only the male was treated, the mating rate was significantly lower at the medium and highest dose when compared to solvent-treated controls (Freeman-Halton test across treatments: p < 0.001, Fig. 4A). Copulations were significantly delayed at all of the tested doses (Kruskal-Wallis test: H = 12.99, p = 0.005, Fig. 5A). Additionally, several typical courtship elements performed by imidacloprid-treated males were delayed in a dose-dependent manner (Fig. S1a–d). If only the female was treated, the mating rate was decreased only at the highest dose (Freeman-Halton test across treatments: p < 0.001, Fig. 4B). However, when copulations did occur under these conditions, already 0.4 ng imidacloprid delayed receptivity signalling by females (Kruskal-Wallis test: H = 9.77 p = 0.021, Fig. 5B) suggesting that the perception of the male aphrodisiac pheromone was impaired in these females. In couples with both partners treated, mating rates decreased at all tested imidacloprid doses by up to 80% (Freeman-Halton test across treatments: p < 0.001, Fig. 4C).

## Discussion

The present study sheds light on a hitherto neglected non-target effect of neonicotinoids by showing that sublethal doses of imidacloprid disrupt the sexual communication in a parasitoid wasp. Virgin *N. vitripennis* females treated with imidacloprid were hampered in their ability to locate the male sex pheromone. Their delayed receptivity signaling during courtship furthermore indicates that they were also less responsive to the male aphrodisiac. Likewise, imidacloprid-treated males were less effective in recognising females and performing courtship suggesting that they were impaired in perceiving the female-derived cuticular hydrocarbons. This might explain why the mating rates of imidacloprid-treated couples decreased.

The use of host-associated chemical cues for host finding was impeded in imidacloprid-treated *N. vitripennis* females. This result and previous findings in a braconid wasp parasitizing lepidopteran larvae^31^ suggest that sublethal neonicotinoid doses affect the host finding ability of parasitic wasps adversely. Mate and host finding are fitness-relevant competencies for parasitoid wasps. Therefore, the effects observed here as well as the decreased fecundity and survival found in other species after exposure to neonicotinoids^2^,^50^, likely compromise the key function of parasitoid wasps as natural enemies in terrestrial ecosystems. More subtle fitness-relevant effects caused by neonicotinoids such as a hampered ability to adjust the optimal offspring sex ratio^45^,^51^ might additionally decrease the reproductive success of parasitoid wasps in the field.

The estimated economic value attributed to biological control by predators and parasitoids in the USA amounts to a total of 4.5 billion US$ per year^26^. Hence, a lower effectiveness of parasitoid wasps caused by sublethal effects of neonicotinoids may not only have severe ecological but also economic consequences. There is no reason to assume that the reported effects on sexual communication and foraging behaviour are restricted to parasitoid wasps. Given that similar effects on the chemical communication will likely be found in other insect taxa, the ecological and economic consequences might be even more severe because of the key role insects play as prey for animals from higher trophic levels^26^. A recently shown correlation between imidacloprid concentrations in the ground water and the decline of insectivorous birds in the Netherlands corroborate these concerns^52^.

An important question is whether the tested doses (0.1–1.1 ng/wasp) are realistic under field conditions. Parasitic wasps may come into contact with sublethal doses of systemic neonicotinoids by feeding on contaminated floral and extrafloral nectar or guttation drops of treated plants. Guttation drops of plants grown from imidacloprid-treated seeds may contain very high levels of the active substance (10–200 ppm^12^). The consumption of a few nanolitres of those guttation drops would result in doses comparable to the ones used here. It is unknown, however, whether parasitoid wasps or other insects consume guttation drops^7^. The neonicotinoid concentrations in nectar from treated plants are highly variable^7^,^14^. An average maximum level in nectar of 1.9 ppb (based on 20 published studies) has been reported^7^ suggesting that this is an unlikely option for parasitoid wasps to ingest the amounts tested here. However, a recent study on *Eucalyptus* trees reported much higher levels (660 ppb), which were shown to be sufficient to cause decreased survival and fitness in four parasitoid species^53^. Furthermore, our results suggest that there is a long-term effect of imidacloprid (Fig. 2B), indicating that detrimental effects may accumulate during long-term exposure to very low doses^54^. Finally, orally consumed neonicotinoids might have even stronger effects than the externally applied doses used here. Exposure of *N. vitripennis* females to 200 ppb imidacloprid (dissolved in sucrose solution), for instance, resulted in 50% mortality after 72 h and impeded optimal sex allocation by the females^45^. The reported average maximum level of neonicotinoids in nectar (1.9 ppb^7^) is approximately 1% of this concentration. In our study, we observed significant effects at 1–5% of the LD_50_ determined for our topical application method. This suggests that exposure of females to contaminated nectar might have similar effects on the chemical orientation of *N. vitripennis* under natural conditions. An alternative way by which parasitoid wasps may take up neonicotinoids is via the host. Juvenile stages of parasitoid wasps may ingest the toxins when feeding on a contaminated host and the same is true for adult females when performing host feeding prior to oviposition^2^. Also, the mere contact of parasitoid wasps with plant leaves that had been treated with systemic neonicotinoids has been shown to cause negative effects on survival of the wasps indicating that a transfer of sublethal doses might be possible by that way^50^.

Many insecticides are neurotoxins that impede the ability of insects to acquire and process information from their environment. Consequently, insecticides other than neonicotinoids, such as organophosphates and pyrethroids, have also been shown to interfere at sublethal doses with the sexual communication and foraging success of insects including parasitoid wasps^55^,^56^,^57^,^58^,^59^,^60^. It therefore seems reasonable to include the study of sublethal effects of insecticides and other anthropogenic substances on the chemical communication of beneficial insects during the approval process for novel substances. The *Nasonia* system is ideal to develop the standardized methods for this pupose, as the pheromones are well-characterised, effective behavioural bioassays are available, and the insects are easily reared in quantity. Thus, the present study not only provides decision makers with important knowledge on hitherto neglected non-target effects of neonicotinoids but also offers tools to study these effects in candidate pesticides to be tested for approval in the future.

## Methods

### Insects

The *N. vitripennis* wasps used in this study originated from the inbred strain Phero01 and were reared on freeze-killed puparia of the green bottle fly *Lucilia caesar* as described previously^35^. To obtain virgin wasps of defined age, parasitoid pupae were excised from host puparia 1–2 days prior to eclosion and kept singly in 1.5 ml microcentrifuge tubes. Each wasp was used only once in the experiments. Treatment with imidacloprid or solvent (control) was performed 0–12 h after eclosion.

### Determination of LD_50_

Acetone solutions (210 nl) containing 0.0, 0.4, 1.1, 2.7, 5.7, 11.8, and 24.0 ng (n = 4 replicates with eight wasps per dose, sex and replicate) imidacloprid were applied to the abdominal tip of cold sedated wasps using a Nanoliter 2010 microinjector mounted to a micromanipulator (World Precision Instruments). Because of the quantitative absorption of the solution through the anal orifice, this approach allowed control of the exact dose taken up by the insects and determination of the LD_50_. Previous studies revealed that low volumes of acetone applied by this method have no visible toxic effects on *N. vitripennis* wasps^61^,^62^,^63^. Mortality was determined after 72 h and the LD_50_ was interpreted from the dose-mortality curve calculated using the polynomial regression of the R package ggplot2^64^.

### Effect of sublethal imidacloprid doses on the female response to the male sex pheromone

The effect of imidacloprid (0.0, 0.1, 0.4 and 1.1 ng/wasp 1 d after treatment; 0.0, 0.4 and 1.1 ng/wasp 3 d after treatment) on the response of females to the male-produced sex pheromone (n = 20 for each treatment) was tested in our well-established two-choice olfactometer (for details see ref. 49). Briefly, 1 μl of the synthetic sex pheromone dissolved in dichloromethane (200 ng/μl RS, 100 ng/μl RR, and 3 ng 4-MQ, synthesized as described in refs 33 and 63) were applied to a disk of filter paper. The ratio of the pheromone components was consistent with the previously reported composition^32^,^33^. Control paper disks were treated with the same amount of pure solvent. After evaporation of the solvent, test and control disks were put into the test and control cavity of the olfactometer and females were released individually into its centre. The time females spent in the test and control cavity was recorded for five minutes using The Observer XT observational software (Noldus, Wageningen, The Netherlands). The olfactometer was turned by 90° after every observation to avoid biased results due to side preferences.

### Effect of sublethal imidacloprid doses on the female response to host odour

The response to host odour by females treated with 0.4 ng imidacloprid and solvent treated control wasps (n = 20) was tested 1 d after treatment using a linear still-air olfactometer described in detail elsewhere^40^. Briefly, the olfactometer consisted of an angular acrylic tube (14 cm × 1 cm × 1 cm) that was divided into two 4-cm test zones at either end and a 5-cm neutral zone in the middle. Five hosts were presented at the end of the test zone and visual contact was prevented by a thin sheet of non-transparent gauze. The control zone was designed identically but left empty. Prior to the test, host volatiles were allowed to diffuse into the olfactometer tube for 5 min. Subsequently, *N. vitripennis* females were released individually into the neutral zone and the time they spent in the test and control zone was recorded for 5 min. The olfactometer was rotated by 180° after every observation to compensate for any unforeseen asymmetry of the set-up.

### Effect of sublethal imidacloprid doses on the courtship behaviour

The courtship behaviour of *N. vitripennis* couples treated with three different sublethal doses of imidacloprid (0.1, 0.4, 1.1 ng per wasp; either males, females or both were treated) and solvent treated control wasps (n = 20 for each treatment) was videotaped 1d after the treatment. Observations lasted until the end of postcopulatory courtship but were terminated after 5 min if couples had not started courtship by that time. Video recordings were analysed using the video module of The Observer XT software. For all observations, the act of copulation was documented and the mating rate for all treatments was calculated. Depending on the treated sex, different elements of the courtship behaviour were also recorded for those couples that did mate. (a) Couples with treated males: (i) time between contact of a male with the female and mounting; (ii) time between receptivity signalling (withdrawing of the antennae by the female) and copulation; (iii) duration of copulation; (iv) time between copulation and remounting the female (after re-mounting, postcopulatory behaviour is shown); (v) total time between start of the observation and copulation. (b) Couples with treated females: (i) time between a male mounting the female and receptivity signalling (lowering of the antennae by the female). This time is correlated to male head nodding duration, a behaviour that is shown by males when applying the aphrodisiac pheromone to the female antennae to elicit receptivity^34^,^37^; (ii) total time spent by a male mounting the female. For couples with both partners treated, only the mating rate was recorded.

### Statistical analyses

Because not all data met the assumptions for parametric statistical analyses, we used non-parametric tests throughout. The sample size for the experiments (n = 20 for all experiments) was calculated using G-power 3.1.9.2 scientific software^65^ considering an α-error of 0.05, a minimum statistical power of 0.8 and effect sizes obtained in similar experiments in previous studies (Cohen’s d ≥0.8). Couples that did not copulate within the observation period of five minutes were excluded from the statistical analysis of all courtship parameters related to copulation (e.g. “time until copulation”). Residence times of female wasps in test and control fields of the olfactometers were compared by Wilcoxon matched-pairs tests. The residence times in the pheromone treated odour field of female wasps treated with different imidacloprid doses were compared by Kruskal-Wallis *H-*tests followed by multiple pairwise Mann-Whitney *U*-tests with sequential Bonferroni correction. Mating rates of imidacloprid-treated wasps were compared by Freeman-Halton tests followed by Fisher’s exact tests with sequential Bonferroni correction for individual comparisons between treated and control wasps. Durations of individual courtship elements in couples with imidacloprid-treated wasps and untreated control couples were compared by Kruskal-Wallis *H-*tests followed by multiple pairwise Mann-Whitney *U*-tests with sequential Bonferroni correction. All statistical analyses were done using Past 3.0 statistical software.

## Additional Information

**How to cite this article:** Tappert, L. *et al*. Sublethal doses of imidacloprid disrupt sexual communication and host finding in a parasitoid wasp. *Sci. Rep.* **7**, 42756; doi: 10.1038/srep42756 (2017).

**Publisher's note:** Springer Nature remains neutral with regard to jurisdictional claims in published maps and institutional affiliations.

## Supplementary Material

Supplementary Figure S1
