# Supplementary material for: Sublethal doses of imidacloprid disrupt sexual communication and host finding in a parasitoid wasp
Source: Sci Rep. 2017 Feb 15;7:42756. doi: 10.1038/srep42756 (PMC5309895; doi:10.1038/srep42756)
Supplement: Supplementary Figure S1 [file srep42756-s1.pdf]

Supplementary data

Sublethal doses of imidacloprid disrupt sexual communication and host finding in a parasitoid wasp

Lars Tappert[1], Tamara Pokorny[1], John Hofferberth[2] & Joachim Ruther[1]*
[1]Institute of Zoology, University of Regensburg, 93053 Regensburg, Germany
[2]Department of Chemistry, Kenyon College, Gambier, OH 43022, USA

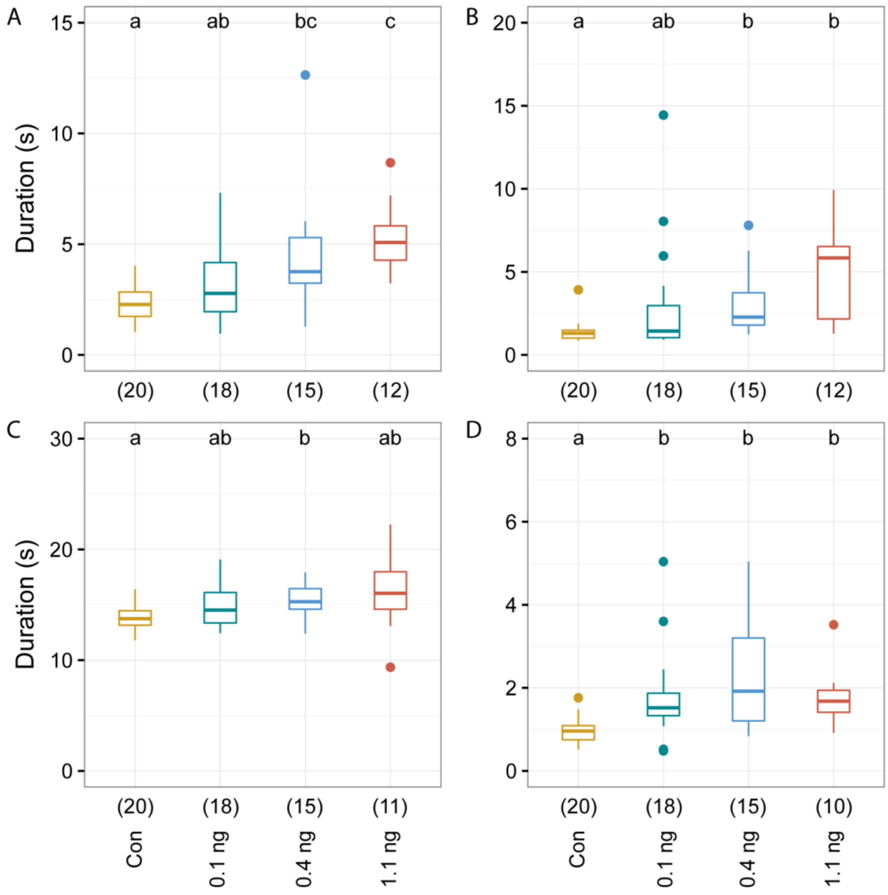

Fig. S1. Effect of sublethal doses of imidacloprid on the courtship behaviour of *N. vitripennis* males. (**A**) time between first contact of a male with the female and mounting; (**B**) time between receptivity signal and copulation; (**C**) duration of copulation; (**D**) time between copulation and remounting the female. Male wasps were treated with a solution of imidacloprid in acetone or the pure solvent (Con). Numbers of replicates (given in parentheses) differ because only those couples that started courtship within 5 min were included. Box-and-whisker plots show median (horizontal line), 25-75% quartiles (box), maximum/minimum range (whiskers) and outliers (> 1.5 × above box height). Statistical analysis by Kruskal-Wallis test followed by multiple pairwise Mann-Whitney *U*-tests with sequential Bonferroni correction. Different lowercase letters within each panel indicate significant differences at $p < 0.05$.
